# Sustainable Ethanol‐Based Reversed‐Phase Liquid Chromatography for Determination of Diltiazem pKa and Quantification with Integrated Green and White Analytical Metrics

**DOI:** 10.1002/open.70215

**Published:** 2026-04-20

**Authors:** Zehra Üstün, İlkay Konçe, Ebru Çubuk Demiralay

**Affiliations:** ^1^ Atayalvac Vocational School of Health Services Suleyman Demirel University Isparta Turkey; ^2^ Faculty of Pharmacy Suleyman Demirel University Isparta Turkey

**Keywords:** diltiazem, EPPI index, green analytical chemistry, *pKa* determination, white analytical chemistry

## Abstract

This study presents a sustainable chromatographic method for the analysis of diltiazem. Retention behavior and the effect of mobile phase pH were investigated to determine the dissociation constant. Chromatographic experiments were performed at 37°C on a Gemini NX C18 column in binary mixtures of ethanol‐water containing 40%, 45%, and 50% (v/v) ethanol and methanol‐water containing 45%, 50%, and 55% (v/v) methanol. Using these retention data, the 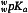 value of diltiazem, which has low solubility in water, was calculated. Beyond analytical performance, the sustainability of method was evaluated multimetrically approach using the Environmental, Performance, and Practicality Index, the Carbon Footprint Reduction Index, and the Sustainability of Analytical Methods Index. This complementary framework provides a holistic assessment of operational greenness, carbon intensity, and global sustainability goals in accordance with the latest guidelines. The method for diltiazem determination according to these tools showed linearity with a correlation coefficient of 0.999 in the concentration range of 1.0–15 µg/mL and achieved acceptable precision and accuracy. Validated according to ICH Q2(R2) guidelines, the procedure was successfully applied to tablet formulations and forced degradation studies. This work establishes a comprehensive, ecofriendly methodological approach suitable for pharmaceutical analysis.

## Graphical abstract

A sustainable reversed‐phase liquid chromatography method is developed for diltiazem analysis and pKadetermination. The study integrates green and white analytical chemistry frameworks to evaluate environmental impact and practicality. The Environmental, Performance, and Practicality Index is utilized as a comprehensive, holistic tool for the sustainability assessment of diltiazem. The method is validated through forced degradation and pharmaceutical analysis.

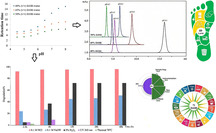

## 1 Introduction

Diltiazem, a benzothiazepine calcium channel blocker, remains an essential agent in cardiovascular medicine. It limits calcium entry through L‐type calcium channels, resulting in vasodilation, reduced myocardial oxygen demand, and improved cardiac performance. Recently, works have emphasized its ongoing clinical relevance and adaptability. The drug is still widely prescribed and has been thoroughly studied for its pharmacokinetic and physicochemical behavior [1, 2, 3]. Clinically, diltiazem shows strong efficacy in treating angina, hypertension, and atrial fibrillation, where it functions as an effective rate control agent [4]. Beyond its cardiovascular role, new evidence points to potential benefits in other therapeutic areas [5, 6]. Studies have reported possible anti‐inflammatory, antiviral, and anticancer properties, suggesting that diltiazem's therapeutic scope is expanding. These findings underscore the pharmacological significance of diltiazem and its refined physicochemical properties in contemporary medicine. Determining the pharmacological and pharmacokinetic data for this widely used active compound is important. Therefore, the primary objective of this study was to experimentally determine the dissociation constant (*pK* *_a_*) of diltiazem using a widely used instrumental method.

Knowing the *pK* *_a_* value of an active pharmaceutical ingredient (API) is highly useful for predicting in vivo absorption, distribution, metabolism, and excretion (ADME) behavior. Although many methods are currently used to determine this physicochemical parameter [7, 8, 9, 10, 11], reverse‐phase liquid chromatography (RPLC) is the preferred method due to its high precision and accuracy. With RPLC, the *pK* *_a_* value is determined by the change in the retention time (*t*_R_) of ionizable compounds in the mobile phase pH at constant column temperature, which depends on the concentration of the organic solvent in the mobile phase and the properties of the stationary phase used. The elution power of the mobile phase in compound analysis depends on the organic solvent used and its concentration. To determine the *pK* *_a_* value, the solubility of the studied compound must first be known. Although water is an environmentally friendly and powerful solvent, it cannot be used alone as the mobile phase in RPLC analysis, especially for hydrophobic compounds. For the RPLC analysis of these analytes, which have low solubility in water, a binary mixture of water and an organic solvent is used. 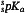 measurements are usually performed in water‐organic solvent mixtures. Methanol (MeOH), acetonitrile (ACN), and tetrahydrofuran (THF) are often preferred in this method, depending on their elution power [12]. The use of large amounts of these organic solvents in RPLC analyses of compounds with low water solubility generates significant waste. To avoid this, environmentally friendly solvents should be preferred. Analyses using environmentally friendly solvents are known as green RPLC [13, 14]. The solvent commonly used today in this green RPLC method is ethanol (EtOH) [14, 15]. EtOH has lower toxicity and vapor pressure than ACN and MeOH. While EtOH and MeOH are similar in terms of chromatographic selectivity, analyte *t*_R_ values can be determined in shorter time periods when analyzed with EtOH than with other toxic solvents [16].

Determining the ionization state of analytes in a specific body compartment relative to the ambient pH provides important data for determining solubility and lipophilicity values. For this purpose, the *pK* *_a_* (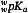) value of diltiazem, which has a low solubility in water (S: 3.11.10^−2^ mg/mL), was also determined in aqueous medium. In this study, the *pK* *_a_* value of diltiazem will be calculated using the *pK* *_a_* (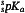) values in water‐EtOH and water‐MeOH binary mixtures and the macroscopic parameters (*X,ε*) of the organic solvent in the mobile phase [17, 18]. In this study, using the capacity factor values of diltiazem at the studied mobile phase pH values, the hydrophobicity descriptor (*φ*_0_) will be calculated for the first time to provide information about the hydrophobicity of the compound [19].

The sustainable analytical methodology necessitates a comprehensive evaluation that transcends traditional greenness assessments by integrating Green Analytical Chemistry (GAC) and White Analytical Chemistry (WAC) frameworks [20]. In this context, a synergistic triplet consisting of the Environmental, Performance, and Practicality Index (EPPI), the Carbon Footprint Reduction Index (CaFRI), and the Sustainability of Analytical Methods Index (SAMI) is utilized to provide a nonredundant and multidimensional characterization [21, 22, 23]. The EPPI serves as the core holistic framework, balancing chromatographic performance with environmental safety by operationalizing the 12 principles of GAC, Green Sample Preparation (GSP), and WAC [24]. To address the critical yet frequently neglected dimension of global warming, CaFRI is employed as a specialized climate‐oriented tool to quantify carbon footprint reduction. Finally, the SAMI is incorporated to align laboratory practices with global environmental targets, evaluating the method's contribution to the United Nations Sustainable Development Goals (SDGs) [23]. This integrated strategy ensures that operational greenness, analytical efficiency, and global sustainability are evaluated through a complementary framework, avoiding the limitations of overlapping single‐metric tools.

For these analytical methods, the method that complies with the GAC and WAC criteria was determined, and optimum chromatographic analysis conditions were decided. The optimized method was validated according to the International Council for Harmonisation of Technical Requirements for Medicines for Human Use (ICH, Q2(R2)) [25] guidelines, and quantitative analysis was performed on a pharmaceutical tablet formulation. Stability studies were also carried out to determine suitable storage conditions for diltiazem.

## 2 Results and Discussion

### 2.1 Retention Behavior and Effect of Mobile Phase Composition

For the analysis of the studied analyte, a modified C18 column was used in this study to overcome the limited pH operating range of conventional silica‐based columns. A Gemini NX C18 (250 x 4.6 mm, 3 µm) column was used for the determination of the 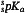 value of the compound. This column features a patented technology in which a polymeric silica layer is grafted onto a pure silica core. This structure, in which ethane ligands are embedded in the bridging polymeric silica, offers exceptional pH stability and mechanical strength [26]. In this study, EtOH and MeOH were selected as organic solvents in the mobile phase for the analysis of analytes with varying polarities. It was not possible to use the same volume percentages for both solvents when determining the liquid chromatographic *t*_R_ value of diltiazem. Due to the stronger elution power of EtOH compared to MeOH, using the same concentrations would have resulted in excessively long retention times with MeOH, which is undesirable for RPLC analysis. Consequently, analyses were carried out using water‐MeOH mixtures (45%, 50%, and 55% v/v MeOH) and water‐EtOH mixtures (40%, 45%, and 50% v/v EtOH) while keeping other chromatographic conditions constant.

**FIGURE 1:**
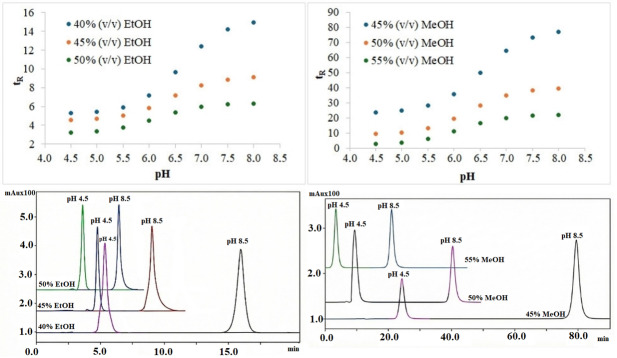
Top—Sigmoidal relationship between *t*_R_ values and pH of the mobile phase for diltiazem in EtOH‐water (left) and MeOH‐water (right) mixtures at 37°C. Bottom—Chromatogram showing the retention times of diltiazem at different pH values in EtOH‐water (left) and MeOH‐water (right) mixtures at 37°C.

In RPLC studies, the 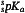 of diltiazem can be determined from the midpoint of sigmoidal curves obtained by plotting the measured t_R_ value against varying mobile phase pH values. The power of mobile phase pH as an optimization tool depends on its dependence on mobile phase composition, necessitating strict control. For this purpose, the mobile phase pH was selected in the study between 4.5 and 8.0. Retention times at each of these pH values were obtained from triplicate analyses. The sigmoidal curves obtained from binary mixtures of EtOH‐water and MeOH‐water are presented in Figure 1. It has been observed that as the pH of the mobile phase increases, the compound is less retained in its ionized form on the RPLC column compared to its molecular form. As the amount of EtOH and MeOH in binary mixtures increases, the elution strength of the mobile phase (decreasing polarity) increases, resulting in faster elution of the compound from the RPLC column. Furthermore, as the pH of the mobile phase increases, the *t*_R_ values of the compounds increase, indicating the ionization behavior of the basic functional group in the chemical structure.

In this study, the 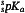 values corresponding to the tertiary amine functional group in the diltiazem structure were determined by the increase in *t*_R_ values as the 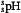 value increased. Since EtOH has a higher elution power (*ε* ^0^ = 3.6) than MeOH (*ε* ^0^ = 3.0), a smaller volume is needed to achieve the same elution power in RPLC [18]. Therefore, the compounds in this study could be analyzed using a mobile phase with a lower EtOH content.

However, the transition from methanol to ethanol involves practical trade‐offs that must be considered. EtOH possesses a significantly higher viscosity compared to methanol, which inherently leads to higher column backpressure. In this study, the use of a Gemini NX C18 column, which utilizes advanced hybrid technology, provided the necessary mechanical strength and durability to withstand these higher pressures without compromising stationary phase integrity. Furthermore, while increased mobile phase viscosity can potentially hinder mass transfer kinetics and reduce chromatographic efficiency, the operation at a controlled temperature of 37°C effectively lowered the viscosity and maintained high theoretical plate counts (*N* ≥ 2000). These technical considerations ensure that the sustainability benefits of ethanol are achieved without sacrificing analytical performance, consistent with recent advancements in green pharmaceutical analysis [27, 28].

The NLREG program was used to process a data set containing experimental *t*_R_ and the corresponding mobile phase conditions (pH and percentages of EtOH and MeOH). The obtained 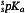 (retention time of the molecular form) and $t_{RBH^{+}}$ (retention time of the ionized form) values, along with their standard deviations, are presented in Table 1.

**TABLE 1:** Data calculated with the NLREG program.

Compounds	Data	Concentration of EtOH			Concentration of MeOH		
		30% (v/v)	35% (v/v)	40% (v/v)	45% (v/v)	40% (v/v)	45% (v/v)
Diltiazem	$t_{RBH^{+}}$	5.193 ± 0.007^a^	4.499 ± 0.043	3.145 ± 0.064	23.256 ± 0.065	8.827 ± 0.016	2.153 ± 0.024
	$t_{RB}$	15.360 ± 0.022	9.197 ± 0.075	6.353 ± 0.019	78.573 ± 0.077	40.088 ± 0.055	22.109 ± 0.034
	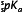	6.610 ± 0.006	6.389 ± 0.044	6.148 ± 0.007	6.530 ± 0.0054	6.300 ± 0.059	6.092 ± 0.081

a Standard deviation.

As shown in Table 1, the 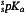 values of diltiazem decreased with increasing EtOH and MeOH in the mobile phase. The 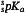 value of the basic functional group decreased by approximately ±0.20 to ±0.25 as EtOH and MeOH concentrations increased. This is attributed to the suppression of specific dissolution by electrostatic interactions. This trend is consistent with the stronger retention of the molecular form of the analyte than the ionized form on the RPLC column due to the change in the pH of the mobile phase [7]. Figure 1 shows the chromatogram showing the change in *t*_R_ values of the molecular and ionized forms of diltiazem in a binary mixture containing EtOH. Figure 1 shows the chromatogram showing the change in retention in MeOH‐water binary mixtures containing MeOH.

Isocratic RPLC is difficult to analyze in 100% water, especially for hydrophobic compounds, and these compounds yield high *t*_R_ values. These *t*_R_ values can be estimated without any experimental work using linear solvent strength theory. This theory assumes that the logarithm of the capacity factor (log*k*) is linearly related to the amount of organic solvent (*φ*) in the binary mixture:

(1) $$\log k=\log k_{w}-S\varphi$$

In this equation, *k*_W_ represents the *k* value of the analyte in the 100% water mobile phase. The solvent strength parameter, S, is a constant whose slope is characteristic of the linear correlation and the specific interaction of the organic modifier with the stationary phase. The *φ* value of the organic solvent varies between 0 and 1 [29, 30].

Diltiazem *k* values were calculated at pH 8.0, where it was retained most on the RPLC column for the studied organic solvent concentrations. A linear relationship was determined between the calculated log*k* values of the compound and the *φ* value for mobile phases containing 40%–50% (v/v) EtOH and 45%–55% (v/v) MeOH (Figure S1).

The hydrophobicity descriptor *φ*_0_, developed by Valkó and Slégel, was obtained using Equation (2) [19].

(2) $$\varphi_{0}=\frac{\log k_{w}}{S}$$

The determination of the hydrophobicity descriptor *φ*_0_ is of paramount importance in pharmaceutical analysis as it correlates directly with the lipophilicity profiles of APIs. Unlike the classical log *p* values, *φ*_0_ derived from linear solvent strength theory provides a dynamic representation of the compound's affinity for the stationary phase relative to the mobile phase composition. For a cardiovascular agent like diltiazem, the *φ*_0_ values calculated in this study (Table S1) indicate its propensity for passive diffusion across lipid bilayers. Recent studies emphasize that *φ*_0_ serves as a more reliable indicator for drug‐membrane interactions than static logP values in pharmaceutical screening [11, 15, 19]. The slight variation in *φ*_0_ between EtOH and MeOH media reflects the differential competitive interactions between the analyte and the organic modifiers for the C18 surface, further validating the necessity of using standardized green solvents for reliable lipophilicity.

### 2.2 Determination of Values and Degree of Ionization of Diltiazem

The pharmacokinetic profile (ADME) of ionizable active pharmaceutical ingredients (APIs) is critically dependent on their aqueous *pK* *_a_* (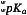) [9]. While these values are determined through both experimental and computational methods, experimentally derived 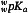 data for the analytes investigated herein are limited. Due to the moderate solubility of the analytes, direct determination of their aqueous *pK* *_a_* values is not feasible. Consequently, we investigated their chromatographic behavior and 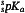 values in various hydroorganic mixtures. The polar protic solvents MeOH and EtOH were selected for this purpose, as they effectively and rapidly dissolve compounds with limited solubility.

Two distinct methodologies were employed to determine the 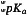 values of diltiazem: one method utilized the *X*_solvent_, while the other was based on the reciprocal of the solvent's dielectric constant (1/*ε*). Using the mole fraction model, the 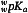 value is determined from the y‐intercept of the linear relationship between the 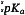 and the *X*_solvent_.

It is essential to distinguish between the 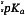, measured directly in the EtOH‐water and MeOH‐water mixtures, and the 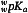. The 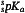 values are inherently influenced by the preferential solvation and the cosolvent's ability to stabilize the ionized or molecular species. To eliminate these solvent‐specific effects and derive the fundamental dissociation constant in a biologically relevant aqueous environment, two complementary thermodynamic models were employed. The mole fraction model (*X*_solvent_) accounts for the nonideal behavior of solvent mixtures, while the dielectric constant model (1/*ε*) addresses the electrostatic work required for charge separation during the ionization process. This dual extrapolation approach ensures that the estimated 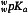 is not merely a mathematical projection but reflects the true protonation equilibrium of the tertiary amine group in diltiazem. These models have been previously validated as robust thermodynamic tools for predicting 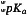 from hydro‐organic measurements [13, 31, 32, 33].

Since mole fraction is not dependent on temperature, the necessary values for the calculations were taken from the literature. Specifically, the *X*_solvent_ for 40%, 45%, and 50% (v/v) EtOH was obtained from Altun [34], and those for 45%, 50%, and 55% (v/v) MeOH were obtained from Canals et al. [35].

**FIGURE 2:**
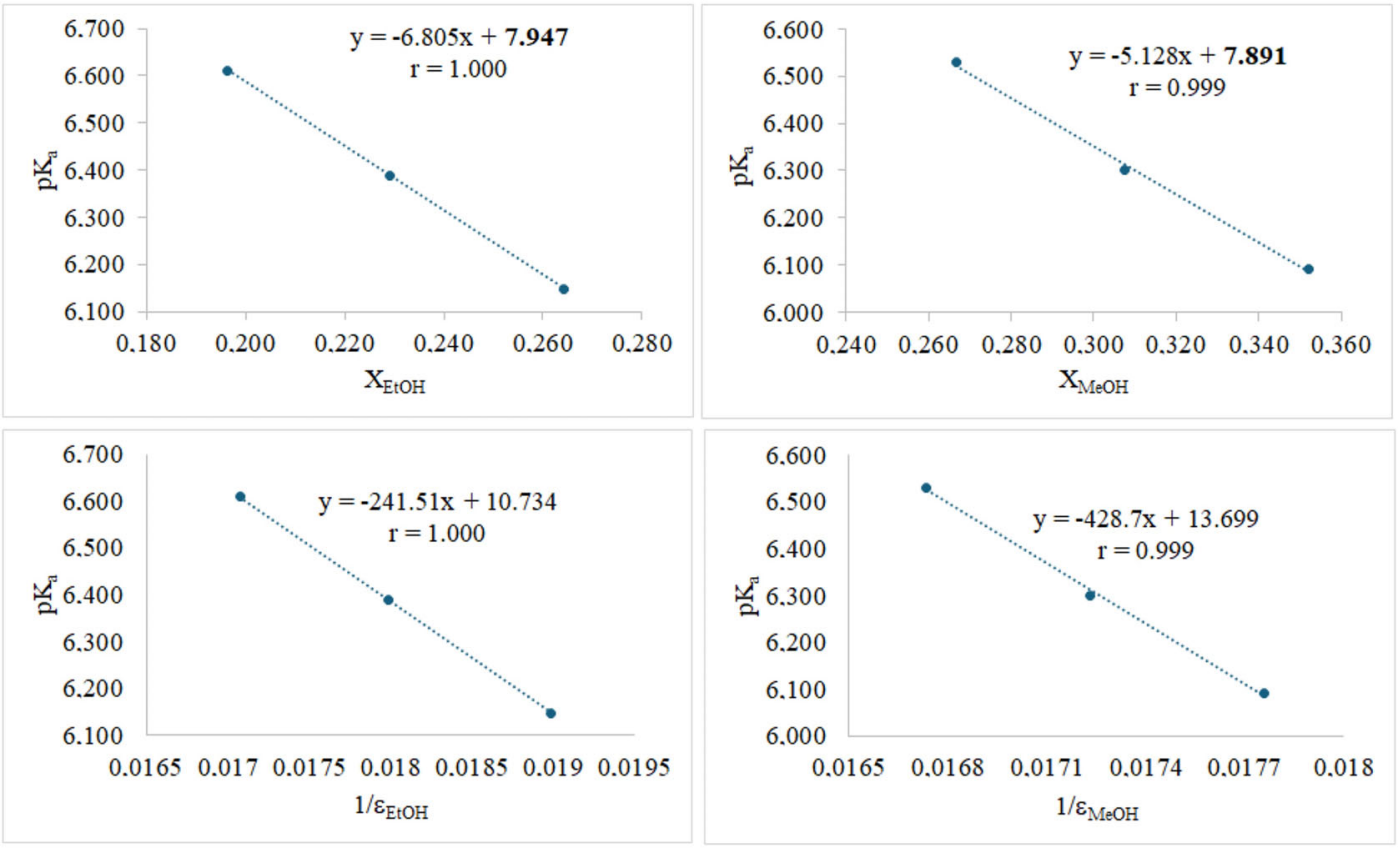
Empirical correlation showing the relationship between *X*_solvent_ ‐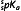 and 1/*ε*_solvent_ ‐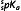.

The linear equations from the mole fraction model are presented in Figure 2. To validate the calculated 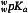 values, a second extrapolation method was used, which correlates the apparent 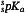 with the reciprocal of the solvent's dielectric constant (1/*ε*). Since the ε value is temperature‐dependent, the values for the solvent mixtures at 37°C were obtained from Albright & Ghosting [36] and Navarkhele & Navarkhele [37]. A linear regression of 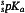 versus 1/*ε* was performed (Figure 2). The 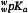 for each compound was then determined by substituting the known dielectric constant of pure water (at 37°C) into the resulting linear equation. A less polar organic co‐solvent decreases the mixture's dielectric constant, which in turn causes the observed shifts in the *pK* *_a_*. The 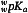 values calculated are depicted in Table 2.

**TABLE 2:** The values of diltiazem in the water medium.

Compound	***X***_**MeOH**_ **‐** 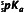	**1/*ε***_**MeOH**_ **‐** 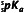	**X**_**EtOH**_ **‐** 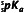	**1/*ε***_**EtOH**_ **‐** 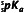
Diltiazem	7.891	7.978	7.947	7.705

**FIGURE 3:**
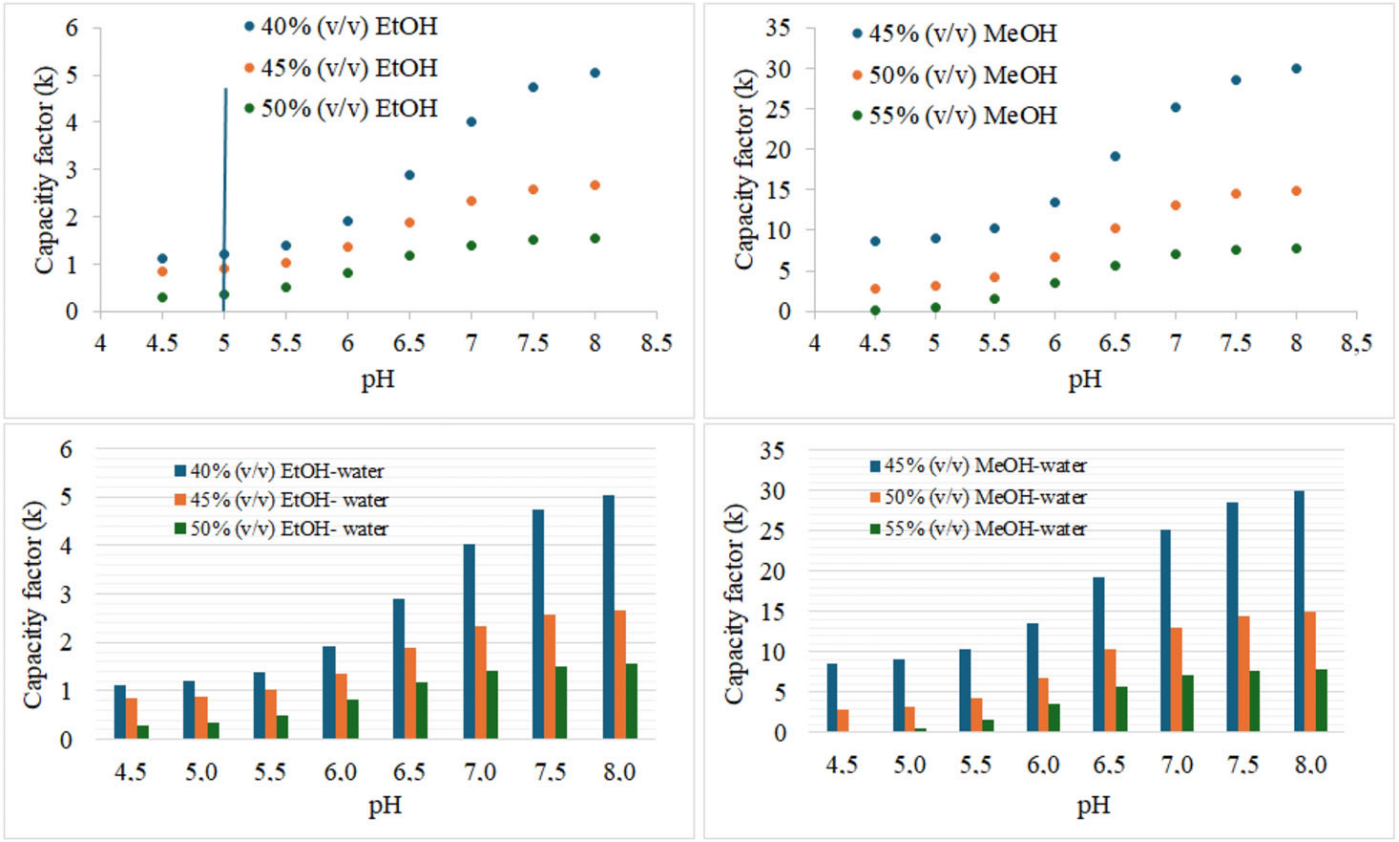
pH‐k relationship in EtOH‐water and MeOH‐water binary mixtures.

Positive slopes in linear functions are characteristic of analytes with basic functional groups. Figure 3 presents the linear relationship between 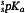 values and *X*_solvent_‐1/*ε*_solvent_. For calculations, the ε value for water at 37°C was taken as 74.94 for MeOH‐water mixtures and 79.72 for EtOH‐water mixtures [36, 37]. Except for minor numerical differences resulting from differences in solvent power, the 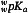 values obtained using both models showed strong agreement and consistency (Table 2).

The 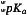 values calculated using two different extrapolation approaches show slight differences. These differences are due to the different polarities of the two cosolvents (0.654 for EtOH; 0.762 for MeOH), leading to differences in compound retention. Consequently, the 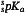 data extrapolated from each solvent system to an aqueous medium differ slightly. There are no experimental studies focusing on determining the *pK* *_a_* values of diltiazem using any analytical method.

A drug's ionization state is a fundamental parameter that directly affects its pharmacokinetic and pharmacodynamic properties. Under physiological pH conditions, the degree of ionization determines the drug's bioavailability and efficiency of reaching target tissues. Therefore, careful consideration of ionization behavior during drug design and formulation development is critical for improving therapeutic efficacy and minimizing side effects. Using the calculated 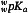 values in this study, diltiazem ionization percentages were calculated over the pH range of 1–12 using the Henderson–Hasselbalch equation [38]. The ionization behavior of the studied analyte is shown in Figure S2. This figure shows that the compounds were ionized to 50% at 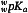 values. Table S2 shows the % ionization data for pH values 1–12 using 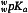 values calculated from EtOH‐water and MeOH‐water binary mixtures. At pH 2 units below its 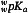, diltiazem is almost completely ionized.

### 2.3 Determination of Optimum Separation Condition

In this study, organic solvents MeOH and EtOH were used in the mobile phase to determine the *pK* *_a_* values of the investigated analytes. Accordingly, the aim was to develop an environmentally friendly RPLC method that can be used for the quantitative determination of the investigated compounds. The optimum liquid chromatographic condition for the determination of diltiazem was determined by determining the average *t*_R_ values at a constant flow rate and column temperature. A *k* value of 1.0 or greater was considered optimal, as was the condition that achieved the shortest determination time. Figure 3 indicates that the EtOH‐water binary mixture containing 40% (v/v) EtOH, adjusted to pH 5.0, was the optimum condition. The chromatogram showing the standard diltiazem peak under these conditions is given in Figure S3. When the *k* values in the MeOH‐water binary mixture were examined, the *k* values were very high in the MeOH‐water binary mixtures containing 45% and 50% (v/v) MeOH. In the MeOH‐water binary mixture containing 55% (v/v) MeOH, the *k* value was above 1.0 starting at pH 6.5. However, under this condition, the consumption of toxic solvents is quite high.

### 2.4 Ecological Impact Assessment

**FIGURE 4:**
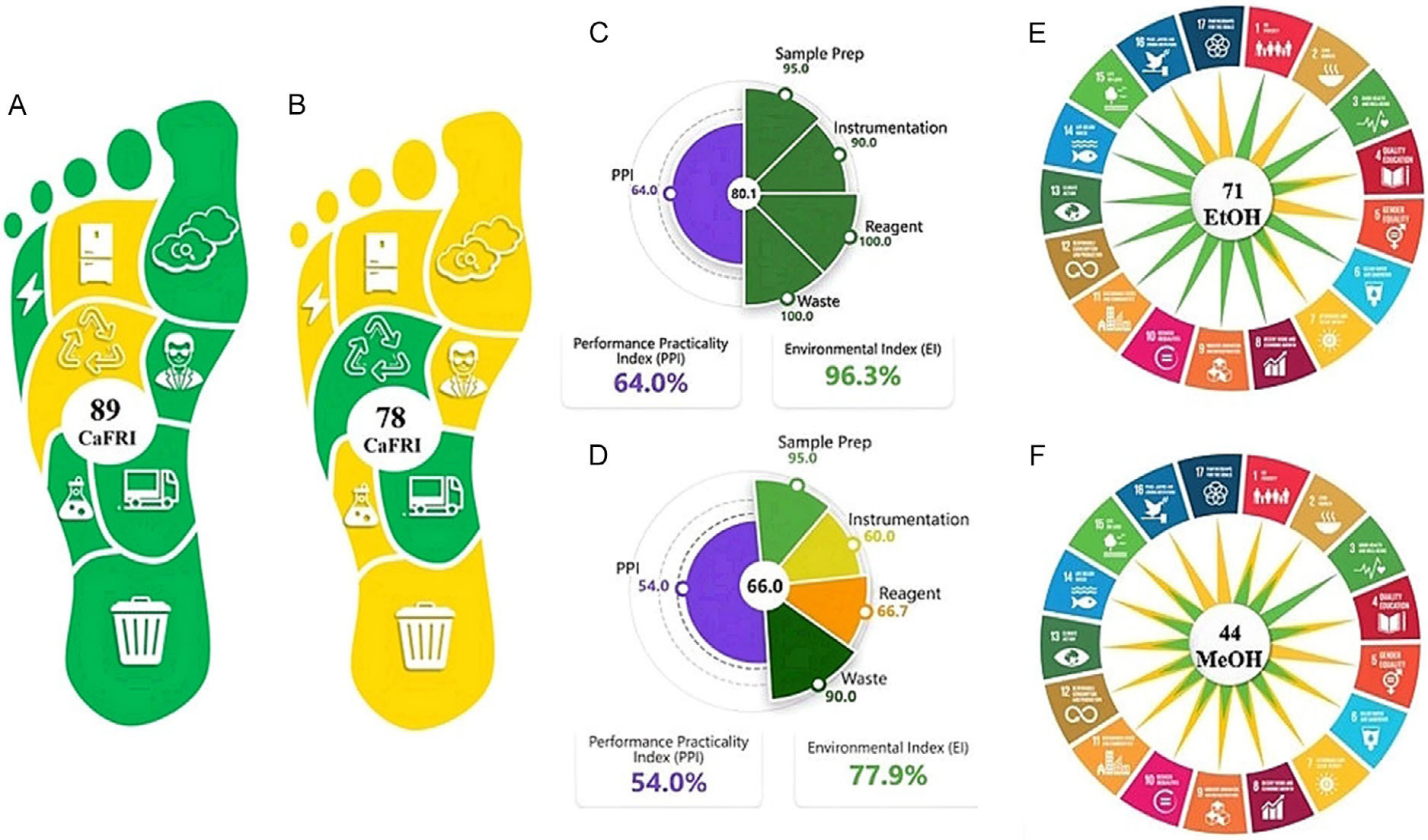
Comparative greenness assessment of the proposed methods using various metric tools. (a,b) CaFRI profiles for A: EtOH (89) and B: MeOH (78). (c,d) EPPI analysis showing PPI and EI values for C: EtOH and D: MeOH. (e,f) SAMI circular plots representing the sustainability alignment for E: EtOH and F: MeOH.

Although there are many tools for assessing greenness in analytical methods, carbon footprint is not considered in the applications used. Therefore, in this study, the CaFRI was used to evaluate the sustainability and greenhouse gas emissions of liquid chromatographic methods developed in hydroorganic solvent environments [22]. This tool provides an assessment of environmental sustainability with parameters related to greenhouse gas emissions. This assessment is a complement to greenness assessment tools. The CAFRI, shown as a pictogram in the shape of a human foot, uses red, yellow, and green colors to indicate low, average, and green ratings, respectively. As shown in Figure 4, the method developed in the EtOH‐water binary mixture, where the numerical score is higher, is more environmentally friendly than the MeOH‐water binary mixture.

The sustainability of the proposed method was validated through a synergistic multimetric framework integrating CaFRI, EPPI, and SAMI (Figure 4). The EtOH‐based approach demonstrated a superior ecological profile with a CaFRI score of 89% and an EPPI(EI) value of 96.3, significantly outperforming the MeOH‐based system (78% and 77.9%, respectively). This distinction is further solidified by the SAMI framework, where the EtOH method achieved “highly sustainable” status with a score of 71, reflecting robust alignment with UN Sustainable Development Goals (SDG 3, 12, and 13) [23]. In contrast, the MeOH system remained at a lower sustainability threshold (SAMI: 44) due to inherent solvent toxicity and waste management limitations. These quantitative metrics, consolidated in Table 3, confirm that transitioning to an EtOH–binary mixture minimizes greenhouse gas emissions while maintaining acceptable operational practicality (PPI: 64.0).

**TABLE 3:** Comprehensive sustainability and performance scores for diltiazem determination for different methods.

Tool, index, and parameters	EtOH method	MeOH method
**EPPI (Total score)**	**80.1**	**66.0**
Environmental impact (EI)	96.3	77.9
Performance and practicality Impact (PPI)	64.0	54.0
**CaFRI (Total score %)**	**89.0**	**78.0**
Energy and equipment	18/20	16/20
Reagents and waste	23/25	15/25
**SAMI (Global sustainability %)**	**71.0**	**44.0**
D1: Environmental (SDG 12, 13)	2 / 2	1 / 2
D2: Health & Safety (SDG 3)	2 / 2	1 / 2
D3: Resource & Energy (SDG 7, 9)	1 / 2	1 / 2
D4: Operational Efficiency (SDG 8)	2 / 2	2 / 2
**Final classification**	**Highly sustainable**	**Sustainable**

The evaluation of analytical sustainability has transitioned from isolated assessments to a more integrated, multimetric framework. As emphasized by Nowak (2025) [39], single‐metric assessments effectively have no mean at all in modern analysis, as they fail to capture the multidimensional nature of sustainable chemistry. Meaningful advancement requires a rigorous multimetric comparison that simultaneously addresses greenness, analytical performance, and operational practicality. As emphasized in the systematic review by Elagamy et al. (2026) [40], relying on a single metric often leads to metric bias where critical aspects like sample preparation or operational practicality are overlooked. The EPPI, developed by Elagamy et al. (2025), stands as the first holistic tool to integrate GAC, GSP, and WAC principles, and its application within this integrated framework is presented here [21]. The EPPI index evaluates the greenness of the method developed by using GAC and GSP principles together. In light of these guidelines, this study presents a complementary and synergistic approach by pairing the EPPI with the CaFRI. EPPI serves as a holistic tool that uniquely integrates the principles of GAC, GSP, and White Analytical Chemistry (WAC) through its Environmental Impact (EI) and Performance and Practicality Impact (PPI) subindices [41]. While EPPI provides an exhaustive profile of operational greenness and method efficiency based on time, cost, accuracy, and resource efficiency, CaFRI adds a vital dimension by quantifying greenhouse gas emissions and energy consumption parameters often omitted in traditional tools like AGREE or GAPI. This pairing aligns with the latest 2025–2026 guidelines [39, 40] for achieving a transparent, nonredundant, and scientifically rigorous sustainability profile in pharmaceutical analysis.

The EPPI index presented in Figure 4 is in the form of a pie chart, and each method is shown with two adjacent halves. Dark green (100–85) indicates the ideal green method. The PPI score on the left is given with a purple gradient. Dark purple (75–100) indicates excellent practicality, and light purple (50–74) indicates acceptable practicality. The value in the middle of this graph gives the total score. Accordingly, it was concluded that the EtOH‐water binary mixture is a green, practical, and efficient method compared to the other medium.

The SAMI represents the most current and comprehensive sustainability metric in the literature [23]. Unlike traditional tools such as AGREE and GAPI, which primarily focus on laboratory greenness, including waste generation, energy consumption, and chemical toxicity, SAMI broadens the evaluative scope by aligning analytical procedures with the United Nations SDGs. This framework assesses a method across four core dimensions: Environmental Responsibility (SDG 12 & 13), focusing on waste management and carbon footprint; Social Impact and Health (SDG 3), evaluating analyst safety and long‐term health effects; Resource and Energy Efficiency (SDG 7 & 9), assessing infrastructure and utility requirements; and Operational Sustainability (SDG 8), regarding cost‐effectiveness and reproducibility. Each criterion is scored on a scale of 0 (poor), 1 (moderate), or 2 (excellent), culminating in a “Sustainability Sun” pictogram that visually summarizes the alignment with 17 different SDGs [23].

The comprehensive sustainability profiles and multimetric scores of both the proposed and conventional methods are summarized in Table 3. According to the EPPI evaluation, the proposed method demonstrated a near‐ideal greenness profile with an EI score of 96.3, significantly surpassing the conventional methanol‐based system (77.9). This performance is a direct result of ethanol's significantly lower toxicity and improved safety profile. The 89% CaFRI score further validates that the proposed method effectively minimizes the carbon footprint by optimizing both solvent selection and operational energy consumption. The most definitive distinction is highlighted by the SAMI scores (71 vs. 44). In accordance with the classification established by Mansour et al. (2026), the EtOH‐based method achieved a score of 71, categorizing it as highly sustainable (≥50) [23]. In contrast, the conventional method remained at 44, falling into the sustainable category but staying below the high‐sustainability threshold due to inherent methanol toxicity (SDG 3) and hazardous waste management challenges (SDG 12/13). These quantitative metrics collectively confirm that the proposed method represents a fundamental advancement in aligning pharmaceutical analysis with global sustainability goals [23].

### 2.5 System Suitability Results

The presented study aimed to provide an environmentally friendly analysis. Quantitative analyses and validation studies were conducted on EtOH‐water binary mixtures containing 40% (v/v) EtOH at pH 5.0. A system suitability test (SST) was performed before each step of method validation [42]. Diltiazem was analyzed in five replicates to determine the k value, tailing factor (TF), theoretical plate count (*N*), retention time, and relative standard deviation (RSD%) for peak area. All calculated parameters were found to be in accordance with the reference parameters. The k value was 1.180 (*k *≥ 1.0), the *N* value was 3862 (*N *≥ 2000), the TF value was 1.54 (TF ≤ 2.0), the RSD% for *t*_R_ was 0.032 (RSD%≤ 1.0), and the %RSD for peak area was 0.639 (RSD%≤ 1.0).

### 2.6 Method Validation Results

The method was evaluated according to the ICH Q2(R2) guideline [25]. The validation process included the assessment of linearity, accuracy, precision, sensitivity, ruggedness, robustness, and stability (forced degradation).

The linearity of the method was determined to be between 1.0 and 15.0 μg/mL. A calibration plot was constructed using peak area (mAu) values obtained from RPLC analysis against varying diltiazem concentrations at seven concentration levels. The results showed excellent correlation (*r *≥ 0.999). The linear regression equation is as follows:

(3) y=(69360.34±788.58)x+(24749±717.64)

The generated calibration data were evaluated using analysis of variance (ANOVA). The *p*‐value for the slope of the linear function is 2.10.10^−13^. This value indicates that the slope is statistically different from zero and that there is a significant linear relationship between concentration and signal. The *p*‐value: 0.284 for the intercept is not significant, supporting the conclusion that the y‐intercepts of the curves are statistically indistinguishable from zero. The statistical significance of the regression was confirmed by ANOVA, yielding an *F*‐value of 2.68.10^8^ and a standard error of the estimate (S*_yx_*) of 53.73. To verify the model reliability, a residual analysis was conducted. The residuals showed a random distribution with no systematic pattern, confirming the homoscedasticity of the variance and the suitability of the ordinary least squares (OLS) model for the quantification of diltiazem in pharmaceutical matrices. These calculated results demonstrate excellent linearity for diltiazem, indicating that the RPLC method can provide reliable and accurate quantitative analysis results. The limits of detection (LOD) and quantitation (LOQ) for diltiazem were calculated based on signal‐to‐noise ratios of 3.3:1 and 10:1, respectively. Accordingly, the LOD and LOQ values were determined as 0.120 and 0.365 μg/mL, respectively [25].

To determine the precision of the RPLC method, intraday and interday reproducibility studies were conducted. For this purpose, diltiazem solutions at two different concentrations within the calibration linear working range were analyzed by RPLC. These prepared independent solutions were analyzed in five replicates within a day. After the intraday analysis, an interday analysis was performed on day 3. These data were evaluated in the calibration function, and the concentration and RSD% values are presented in Table S3. Satisfactory results with RSD% less than 2% were obtained. These data comply with the ICH Q2 (R2) guideline, and the method has high precision [25].

As described in the Materials and Methods section, a tablet solution sample containing the active ingredient diltiazem (Diltizem, Gensenta, 30 mg Diltiazem) was prepared. The amount of diltiazem in the tablet samples was calculated using the peak area values obtained from repeated high‐performance liquid chromatography (HPLC) analyses. The data are presented in Table 4.

**TABLE 4:** The amount of diltiazem in the tablet sample and the recovery results.

Parameters	Results
Labeled claim (mg)	30.00
Amount found (mg)^a^	29.82
RSD%	0.23
Bias%	−0.61
Recovery%	99.39
RSD%	0.23
Bias%	−0.61

a Five experiments.

The data obtained showed that the amount of diltiazem in the tablet sample analyzed was very close to the amount indicated on the tablet label. Since the calculated RSD% (relative error) was less than 1%, it was concluded that the sensitivity and accuracy of the results were good. A recovery study was conducted to determine the accuracy of the method. Diltiazem standard solution (5 µg/mL) was added to the tablet sample in a way that did not exceed the calibration linear range, and the average recovery value of the method was calculated (Table 4). This quantitative analysis study concluded that the commonly used excipients in tablets did not affect the analysis results. Chromatograms showing the tablet sample and the tablet sample with a specific concentration of diltiazem added are given in Figure S4. No interference peaks were observed in the chromatograms.

The ruggedness of the developed sustainable method was evaluated by performing five replicate analyses of diltiazem solution (7.5 µg/mL) prepared on the same day under the same conditions with different analysts. According to the results obtained, the calculation of pooled RSD values less than 3% confirmed the ruggedness of this RPLC method (Table S4).

For robustness tests, which indicate that the developed method remains unaffected by minor changes, the flow rate, mobile phase pH, EtOH amount in the mobile phase, and column temperature were varied. Each condition was analyzed by the same person. Table S5 shows that these changes did not have a significant effect on the peak areas of diltiazem. These results demonstrate that the developed method is robust.

**FIGURE 5:**
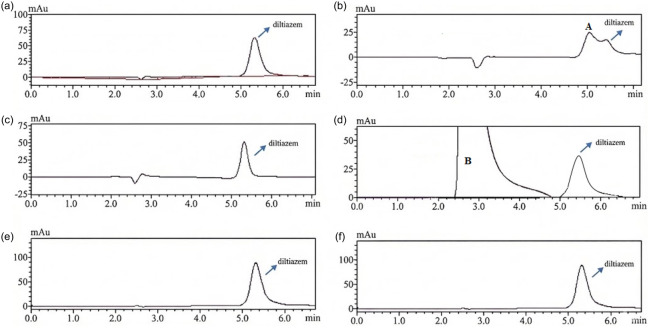
HPLC chromatograms of diltiazem after different stress conditions: (a) nondegraded, (b) acidic degradation, (c) alkaline degradation, (d) oxidative degradation, (e) photolytic degradation, and (f) thermal degradation.

The stability of an active ingredient must be determined because the resulting degradation products can reduce the effectiveness of the drug and cause side effects. Stability studies are conducted to identify the degradation products formed under different conditions (chemical/physical). The analysis performed with the method developed in this study is the first of its kind. Forced degradation studies were performed to evaluate the chemical stability of diltiazem under acidic, basic, oxidative, photolytic, and thermal stress conditions. The degradation percentage was determined by comparing the peak area of the stressed samples with that of the initial undegraded solution and expressing the loss in peak area as a percentage of the undegraded peak area. All stress experiments were conducted under controlled conditions, and the applied parameters are summarized in Table S6. The applied stress conditions were described in detail in the Experimental section. Figure 5 shows representative chromatograms of diltiazem under various stress conditions.

As shown in the chromatograms obtained in Figure 5, diltiazem was observed to be stable and unaffected by photolysis and thermal degradation. Under alkaline and oxidative conditions, it showed little degradation at 2 and 6 h. However, under acidic hydrolysis, significant degradation (91.378%) was observed from the 2nd hour onward, and a distinct degradation product (A) was formed at 5 min. Diltiazem showed 36% oxidative degradation from the 12th hour onward, and a degradation product (B) was formed at 3 min. Table 5 presents the percentage degradation of diltiazem.

**TABLE 5:** Results of forced degradation studies under various stress conditions.

Stress Condition	Time (h)	Degradation (%)
0.1 M HCl (acidic)	2	91.878
	6	95.131
	12	95.141
	24	96.001
	48	98.704
0.1 M NaOH (basic)	2	29.116
	6	26.806
	12	41.735
	24	45.012
	48	50.881
3% H_2_O_2_ (oxidative)	2	5.002
	6	34.503
	12	36.925
	24	51.661
	48	72.207
UV 365 nm (photolytic)	3	3.090
	6	5.978
Thermal (70°C)	3	1.507

A detailed literature review was conducted for validation studies on the liquid chromatographic determination of diltiazem, and it was observed that C8 columns were rarely preferred in the analyses, while C18 columns were predominantly preferred. In general, mobile phases containing varying ratios of ACN or MeOH were used [43, 44, 45, 46, 47, 48, 49, 50, 51, 52]. In addition to all these studies, the study by Sadeghi et al. (2013) can be shown as the only study in the literature as an environmentally friendly analysis [51]. Diltiazem in topical preparations was determined on a C18 column using a mobile phase consisting of an ethanol‐phosphate buffer (35:65, v/v) mixture. This study, like other studies using toxic solvents, was evaluated in terms of validation parameters such as linearity, accuracy, sensitivity, specificity, robustness, and forced degradation. No method optimization or mobile phase pH standardization was performed in this study. Methods developed for diltiazem have been successfully applied to various matrices such as tablets, capsules, and human serum, as well as more complex formulations such as topical gels and nanoethosomes. There is only one study in the literature where chromatographic conditions were optimized using advanced statistical approaches such as Analytical Quality Design (AQbD) and Box‐Behnken design instead of classical trial‐and‐error methods [52]. In this study, the quantitative determination of diltiazem was performed in an aqueous mobile phase containing 50% MeOH without any control. In addition, the stability of the compound was investigated under normal and stressful conditions. Accordingly, the highest degradation was observed under alkaline conditions (24.89%), and the lowest under thermal conditions (3.84%).

## 3 Conclusion

This study presents a comprehensive, environmentally friendly chromatographic strategy for determining the dissociation constant of diltiazem using the RPLC method within an integrated green and white analytical chemistry framework. This research, developed to determine 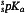 values in EtOH:water and MeOH:water binary mixtures as well as in aqueous medium, represents a first in the literature. The proposed EtOH‐based RPLC method significantly minimized solvent toxicity and carbon intensity compared to conventional systems. The sustainability of the method was rigorously characterized through a synergistic triplet of complementary tools: EPPI, CaFRI, and SAMI. The SAMI results provided a definitive quantitative distinction, where the EtOH‐based method achieved a highly sustainable score of 71, significantly exceeding the 50‐point threshold established for global sustainability alignment, whereas the conventional MeOH–based mixture remained at 44. The CaFRI (89.0%) further confirmed the method's excellence in minimizing greenhouse gas emissions and operational energy consumption. Furthermore, the EPPI index, utilized as a core holistic framework in this study, demonstrated the superior analytical performance and environmental applicability of the EtOH–water medium. These findings confirm that the proposed strategy offers a nonredundant and scientifically rigorous alternative to conventional systems, effectively aligning pharmaceutical analysis with both chromatographic efficiency and international sustainability goals.

The method was validated according to the ICH guidelines under the determined optimum conditions for qualitative and quantitative determination of the compound. For intraday and interday reproducibility studies, RSD% values were calculated to be below 2.0%. Accuracy studies yielded high recovery rates within acceptable limits of 98%–102%. The robustness of the method was verified by varying parameters such as mobile phase organic solvent content, flow rate, column temperature, and mobile phase pH. The robustness of the developed sustainable method was evaluated by different analysts under the same conditions on the same day, concluding that it was suitable. Forced degradation studies determined the stability of the compound under different conditions. In conclusion, this environmentally friendly method developed for diltiazem is suitable for routine analysis and quality control studies.

## 4 Experimental

### 4.1 Chemicals and Reagents

All chemicals were of analytical reagent grade and were used as received without additional purification. Diltiazem hydrochloride (≥98%) was obtained from Santa Cruz Biotechnology (USA) and used as the reference standard. The commercial pharmaceutical formulation used for quantification and recovery studies was Gensenta Diltiazem tablets (30 mg). EtOH (≥99.9%) and MeOH (≥99.9%), both of HPLC grade, were purchased from Isolab (Germany). Sodium hydroxide (NaOH), hydrochloric acid (HCl), hydrogen peroxide (35%, H_2_O_2_), o‐ phosphoric acid (o‐H_3_PO_4_), potassium bromide (KBr), and potassium hydrogen phthalate (KHP) of analytical grade were obtained from Merck (Darmstadt, Germany). Ultrapure water was used throughout the study for the preparation of mobile phases, standards, and sample solutions.

### 4.2 Instrumentation and Chromatographic Conditions

All chromatographic analyses were carried out using a Shimadzu i‐Series HPLC system (Model LC‐2050C 3D, Kyoto, Japan) equipped with a quaternary solvent delivery unit, an autosampler, a column oven with forced‐air circulation, and a photodiode array (PDA) detector. The system was controlled by LabSolutions software and operated under fully automated conditions. Chromatographic determinations were performed on a Gemini NX C18 column (250 x 4.6 mm I.D., 5 µm; Phenomenex, USA). All chromatographic measurements were conducted at 37°C to examine temperature‐dependent retention changes. The flow rate was maintained at 1.0 mL/min, and the detection wavelength was 236 nm. Between analyses, the column was equilibrated for at least 40 min with the corresponding mobile phase until baseline stabilization. To ensure reproducibility, all experiments were conducted in triplicate, and the mean retention time (*t*_R_) was used for further calculations. The average *t*_R_ was calculated from these replicates, showing a precision with an RSD% of approximately 0.01.

To determine the 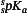 values of diltiazem, different mobile phase compositions were tested under green chromatographic conditions using binary solvent systems of EtOH‐water and MeOH‐water. In the study, diltiazem *t*_R_ values in binary mixtures of EtOH‐water containing 40%, 45%, and 50% (v/v) EtOH and MeOH‐water containing 45%, 50%, and 55% (v/v) MeOH were determined by triplicate analyses. The mobile phases were buffered with 30 mM o‐H_3_PO_4_, and pH adjustments were performed with 1 M NaOH as previously established by our group [31]. The effective pH range examined was 4.5–8.0. The pH values of the binary mixtures in this mobile phase were evaluated according to the International Union of Pure and Applied Chemistry (IUPAC) guideline, considering the reference pH values obtained from the National Institute of Standards and Technology (NIST) [53, 54, 55] KHP (0.05 mol/kg) was used as a reference standard for electrode calibration in hydroorganic mixtures [31].

#### 4.2.1 Capacity Factor Determination

The capacity factor (*k*) is calculated using *t*_R_, the analyte retention time, and *t*_0_, the column dead time. *t*_0_ was determined experimentally using uracil (0.01%, w/v). To minimize random errors, the average *t*_Rs_ of both analytes and uracil were obtained from three replicate injections.

### 4.3 Preparation of Solutions and Tablet Formulation

In all chromatographic experiments, potassium bromide (KBr) was used as a nonretained compound (*t*_0_ marker) to determine the column dead time and calculate retention factors (*k*). To calculate the k value of the studied compounds, use the *t*_0_ value of the KBr solution (0.01%, w/v). Diltiazem stock solution was prepared as 50 μg/mL in the studied mobile phase medium. Before analysis, the mobile phases were filtered and degassed by ultrasonication to ensure baseline stability and reproducible results.

For quantitative analysis of the diltiazem‐containing tablet formulation, ten tablets containing the active ingredient were crushed in a porcelain mortar. Tablet powder equivalent to one tablet was weighed and placed in a volumetric flask (250 mL). The solid was dissolved in an ultrasonic bath by adding the mobile phase, and the volume was adjusted to the desired volume. This aqueous solution was filtered through blue‐band filter paper and diluted to different concentrations to ensure the diltiazem concentration remained within the linear calibration range.

### 4.4 Determination of Data

Chromatographic retention data were analyzed using the NLREG software package [56] to perform nonlinear least‐squares regression based on a Henderson–Hasselbalch model. This approach correlates the pH‐dependent retention behavior of analytes with their degree of ionization and enables estimation of 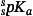 values. The theoretical function minimizes the difference between experimental and calculated capacity factors through iterative optimization, following the methodology described by Rosés & Bosch and Daldal & Çubuk Demiralay [32, 33].

### 4.5 System Suitability

System suitability tests (SST) were performed to demonstrate the overall performance of the liquid chromatographic system and to demonstrate that the method can provide accurate and reproducible results under defined experimental conditions. SST was performed at a concentration of 7.5 µg/mL (which lies within the calibration range) by injecting the standard solution under the determined chromatographic conditions. The resulting *t*_R_, peak areas, theoretical plate number (*N*), TF, and k values were calculated, and the RSD% for *t*_R_ and peak area were computed to affirm system performance in line with ICH guidelines [25].

### 4.6 Method Validation

The RPLC method was validated according to the ICH guidelines Q2(R2) on analytical method validation [25]. The validation process included the assessment of linearity, accuracy, precision, sensitivity, robustness, and stability (forced degradation).

#### 4.6.1 Studies on Determining the Linear Range

To determine the linearity range of the optimized method under optimal chromatographic conditions, a calibration study was conducted by preparing standard diltiazem solutions in the concentration range of 1–15 µg/mL. For this purpose, the average peak areas obtained at the studied concentration values were used. The regression equation and correlation coefficient (*r*) were determined [25]. The sensitivity of the developed method was determined by the LOD and limit of quantification (LOQ).

#### 4.6.2 Precision and Accuracy Studies

The precision of the method was determined at two concentration levels (2 and 13 µg/mL). Mean concentration values and %RSD were calculated for intra‐ and interday reproducibility. To determine accuracy, the percent recovery was determined by adding diltiazem standard solution to the prepared tablet solution [25].

#### 4.6.3 Ruggedness

The ruggedness of the developed sustainable method was evaluated by performing five replicate analyses of diltiazem solution (7.5 µg/mL) prepared on the same day under the same conditions with different analysts.

#### 4.6.4 Robustness

The robustness of the developed RPLC method was assessed to assess its reliability under minor modifications. All robustness experiments were carried out at a concentration level of 13 μg/mL. For this purpose, mobile phase organic solvent content (35%–45% v/v EtOH), flow rate (0.8–1.2 mL/min), column temperature (25°C–45°C), and mobile phase pH (4.0–6.0) were selected as test parameters. The effects of these modifications on t_R_ and peak area were determined.

#### 4.6.5 Specificity

The stability of the developed RPLC method was investigated under various stress conditions, including acidic, basic, oxidative, photolytic, and thermal degradation.

### 4.7 Safety and Waste Disposal

All waste solvents were collected separately according to their composition (MeOH and EtOH mixtures) and disposed of in accordance with institutional and ISO 14001 environmental safety protocols. The substitution of EtOH as a primary organic modifier significantly reduced the volume of hazardous waste generated. EtOH‐based mobile phases were neutralized and disposed of *via* aqueous treatment streams, consistent with ISO 14001 guidelines [57, 58].

### 4.8 Greenness and Whiteness Evaluation of the Optimum Analysis Method

The suitability of the developed RPLC methods for GAC and WAC was evaluated with CAFRI, EPPI, and SAMI tools [21, 22, 23].

## Supporting Information

Additional supporting information can be found online in the Supporting Information section.

## Funding

This study was supported by the Scientific Research Projects Coordination Unit of Süleyman Demirel University under project number TAB‐2024‐9378.

## Conflicts of Interest

The authors declare no conflicts of interest.

## Supporting information

Supplementary Material

## Data Availability

The data that supports the findings of this study are available in the Supporting Information of this article.

## References

[1] D. Stepanovs , M. Jure , M. Gosteva , J. Popelis , G. Kiseļovs , and A. Mishnev , “Crystal Structures and Physicochemical Properties of Diltiazem Base and Its Acetylsalicylate, Nicotinate and L‐Malate Salts,” CrystEngComm 18 (2016): 1235–1241.

[2] L. Diniz , C. Franco , D. Silva , et al., “Multicomponent Ionic Crystals of Diltiazem with Dicarboxylic Acids toward Understanding the Structural Aspects Driving the Drug‐Release,” International Journal of Pharmaceutics 602 (2021): 120790. 10.1016/j.ijpharm.2021.120790 34116180

[3] S. Jaiswal , K. Yadav , S. Shukla , and R. Mitra , “Formulation, Optimization, and Evaluation of Solid‐Lipid Formulation for Bioavailability Enhancement Utilizing Diltiazem Hydrochloride,” Current Applied Materials 03 (2024): e26667312325351.

[4] J. Singh , A. Elton , and M. Kwa , “Comparison of Various Calcium Antagonist on Vasospastic Angina: A Systematic Review,” Open Heart 10 (2023): e002179. 36634997 10.1136/openhrt-2022-002179 PMC9843173

[5] R. Kerep , T. Šeba , V. Borko , et al., “Potential Clinically Relevant Effects of Sialylation on Human Serum AAG‐Drug Interactions Assessed by Isothermal Titration Calorimetry: Insight into Pharmacoglycomics?,” International Journal of Molecular Sciences 24 (2023): 8472. 37239819 10.3390/ijms24108472 PMC10218007

[6] J. Zhang , L. Liu , C. Liu , M. Han , C. Xu , and R. Qiu , “Diltiazem Is a Useful and Effective Medication for Reversal of Coronary Artery Spasm‐Induced Complete Atrioventricular Block: A Case Report,” Frontiers in Cardiovascular Medicine 10 (2023): 1134658. 37077742 10.3389/fcvm.2023.1134658 PMC10106591

[7] Y. Kazakevich and R. Lobrutto , HPLC for Pharmaceutical Scientists (John Wiley & Sons, 2007).

[8] V. R. Meyer , Practical High‐Performance Liquid Chromatography (John Wiley & Sons, 2010).

[9] F. Kalkir , E.Çubuk Demiralay , Y. D. Daldal , and H. Yılmaz , “Simultaneous Determination of Loratadine and Pseudoephedrine Sulfate in Pharmaceutical Formulation by LC–MS/MS Method: Development, Validation, and Application to Stability Studies,” Journal of Pharmaceutical and Biomedical Analysis 256 (2025): 116671.39818020

[10] J. Reijenga , A. van Hoof , A. van Loon , and B. Teunissen , “Development of Methods for the Determination of pKa by Capillary Electrophoresis,” Analytical Chemistry Insights 8 (2013): 53–71. 23997574 10.4137/ACI.S12304 PMC3747999

[11] Z. Üstün and E.Ç. Demiralay , “Determination of the Dissociation Constants of Six Pharmacologically Active Imidazole Antifungal Agents by RPLC Method,” Microchemical Journal 219 (2025): 116019.

[12] V. Ganesh , P. Poorna Basuri , K. Sahini , and C. N. Nalini , “Retention Behaviour of Analytes in Reversed‐Phase High‐Performance Liquid Chromatography‐ A Review,” Biomedical Chromatography 37 (2023): e5482. 35962484 10.1002/bmc.5482

[13] Y. D. Daldal , “Switch from the Traditional RPLC Method to the Green RPLC Methods in the Determination of the Ionization Constant (pKa) of Favipiravir,” ChemistrySelect 9 (2024): e202402900.

[14] M. Yabré , L. Ferey , I. T. Somé , and K. Gaudin , “Greening Reversed‐Phase Liquid Chromatography Methods Using Alternative Solvents for Pharmaceutical Analysis,” Molecules 23 (2018): 1065. 29724076 10.3390/molecules23051065 PMC6100308

[15] İ. Konçe and E.Ç. Demiralay , “Use of Green Analysis Methods to Determine the Ionization Constant Values of Isoxazolyl Penicillins,” Journal of Molecular Liquids 392 (2023): 123306.

[16] L. R. Snyder , J. J. Kirkland , and J. L. Glajch , Practical HPLC Method Development, 2nd ed. (John Wiley & Sons, 1997).

[17] R. Bergés , V. Sanz‐Nebot , and J. Barbosa , “Modelling Retention in Liquid Chromatography as a Function of Solvent Composition and pH of the Mobile Phase,” Journal of Chromatography A 869 (2000): 27–39. 10720222 10.1016/s0021-9673(99)00915-2

[18] C. F. Poole and S. K. Poole , Chromatography Today (Elsevier Science, 1991).

[19] K. Valkó and P. Slégel , “New Chromatographic Hydrophobicity Index Based on the Slope and the Intercept of the Log k′ versus Organic Phase Concentration Plot,” Journal of Chromatography A 631 (1993): 49–61.

[20] M. S. Rahman , W. Hiers , A. Davidson , C. Chabot , F. Riley , and M. Coutant , “Green and White Analytical Chemistry: Advances, Comparisons, Future Perspectives, and a Proposal for Green Financing Model for Analytical Chemistry,” Journal of Pharmaceutical Sciences 114 (2025): 104005. 40998048 10.1016/j.xphs.2025.104005

[21] S. H. Elagamy , K. K. P. Kannaiah , H. K. Chanduluru , N. Yahaya , and R. H. Obaydo , “EPPI: A Comprehensive Index Framework for Assessment of Sustainability Performance, and Practicality of Analytical Methods,” Green Analytical Chemistry 15 (2025): 100306.

[22] F. R. Mansour and P. M. Nowak , “Introducing the Carbon Footprint Reduction Index (CaFRI) as a Software‐Supported Tool for Greener Laboratories in Chemical Analysis,” BMC Chemistry 19 (2025): 121. 40346688 10.1186/s13065-025-01486-2 PMC12065229

[23] F. R. Mansour , M. Locatelli , and A. Bedair , “Sustainability of Analytical Methods Index (SAMI) as an SDG‐Based Tool for Chemical Analysis,” Sustainable Chemistry and Pharmacy 49 (2026): 102294.

[24] A. E. F. Abbas , G. Magdy , and M. K. Halim , “MA Tool—Multi‐Color Assessment Platform for White Analytical Chemistry: Unified Evaluation of Method Greenness, Practicality, Performance, and Innovation,” Microchemical Journal 216 (2025): 114781.

[25] International Council for Harmonisation (ICH) , ICH Harmonised Guideline: Q2(R2) – Validation of Analytical Procedures, 2023.

[26] E. M. Borges and D. A. Volmer , “Critical Review of Current and Future Trends in Environmental and Pharmaceutical Analysis,” Journal of Chromatographic Science 53 (2015): 1107–1122. 25609601 10.1093/chromsci/bmu173

[27] R. Ikhar , P. Sabale , and V. Sabale , “Development of Green Spectrofluorimetric Method for Quantitative Estimation of Favipiravir in Human Plasma Using Application of Multi‐Color Assessment Tool,” Microchemical Journal 224 (2026): 117560.

[28] R. Ikhar , H. Bele , P. Sabale , A. Warokar , and V. Sabale , “Development of Stability‐Indicating Green HPTLC Method for Quantitative Analysis of Baricitinib in API and Pharmaceutical Formulation,” Journal of Pharmaceutical Innovation 21, no. 3 (2026): 217.

[29] A. Carotti , I. Varfaj , I. Pruscini , et al., “Estimating the Hydrophobicity Extent of Molecular Fragments Using Reversed‐Phase Liquid Chromatography,” Journal of Separation Science 46 (2023): 2300346. 10.1002/jssc.202300346 37438993

[30] C. F. Poole and S. N. Atapattu , “Determination of Physicochemical Properties of Small Molecules by Reversed‐Phase Liquid Chromatography,” Journal of Chromatography A 2020 (1626): 461427. 10.1016/j.chroma.2020.461427 32739066

[31] Z. Öztürk , E.Çubuk Demiralay , and H. Yilmaz , “Determination of Retention Behavior and pKa Values of Some Phenothiazines with Green Chemistry Approach,” ACS Omega 10 (2025): 51050–51060. 41210795 10.1021/acsomega.5c05625 PMC12593965

[32] M. Rosés and E. Bosch , “Influence of Mobile Phase Acid–base Equilibria on the Chromatographic Behaviour of Protolytic Compounds,” Journal of Chromatography A 982 (2002): 1–30. 12489853 10.1016/s0021-9673(02)01444-9

[33] Y. D. Daldal and E.Çubuk Demiralay , “Chromatographic and UV–visible Spectrophotometric pKa Determination of Some Purine Antimetabolites,” Journal of Molecular Liquids 317 (2020): 113930.

[34] Y. Altun , “Study of Solvent Composition Effects on the Protonation Equilibria of Various Anilines by Multiple Linear Regression and Factor Analysis,” Journal of Solution Chemistry 33 (2004): 479–497.

[35] I. Canals , F. Z. Oumada , M. Rosés , and E. Bosch , “Retention of Ionizable Compounds on HPLC. 6. pH Measurements with the Glass Electrode in Methanol‐Water Mixtures,” Journal of Chromatography A 911 (2001): 191–202. 11293580 10.1016/s0021-9673(00)01271-1

[36] S. Albright and L. S. Gosting , “Dielectric Constants of the Methanol‐Water System from 5° to 55°,” Journal of the American Chemical Society 68 (1946): 1061–1063. 20985620 10.1021/ja01210a043

[37] V. Navarkhele and A. Navarkhele , “Static Dielectric Constants, Densities, Refractive Indices and Related Properties of Binary Mixtures at Various Temperatures Under Atmospheric Pressure,” International Journal of Thermodynamics 25 (2022): 1–10.

[38] R. J. Tallarida and R. B. Murray “ Henderson‐Hasselbalch Equation,” in Manual of Pharmacologic Calculations (Springer, 1987), 74–75.

[39] P. M. Nowak , “How to Correctly Evaluate Greenness, Whiteness and Other “Colours”? Introducing General Rules of a Good Evaluation Practice,” Green Chemistry 27 (2025): 6699–6710.

[40] S. H. Elagamy , A. Fuente‐Ballesteros , H. K. Chanduluru , and R. H. Obaydo , “Systematic Review of Recent Metrics (2020‐2025) for Greenness, Applicability, and Analytical Performance with Guidelines for Practical use,” Results in Chemistry 20 (2026): 103048.

[41] D. Pingili , A. Awasthi , and M. M. Varshney , “White Analytical Chemistry: A Review of Current Developments,” Journal of Analytical Chemistry 80 (2025): 959–968.

[42] M. Wells and M. Dantus , “Validation of Chromatographic Methods,” in Analytical Instrumentation Handbook (CRC Press, 2004), 1015–1033.

[43] N. Sultana , M. S. Arayne , N. Shafi , F. A. Siddiqui , and A. Hussain , “Development and Validation of New Assay Method for the Simultaneous Analysis of Diltiazem, Metformin, Pioglitazone and Rosiglitazone by RP‐HPLC,” Journal of Pharmaceutical Sciences 49, no. 10 (2024), 10.1093/chrsci/49.10.774. 22080805

[44] S. Patel , A. Patel , C. Chandarana , P. Bhavesh , P. Mehul , and D. Shah , “Critical Insights into Analytical Methodologies for Lidocaine Hydrochloride and Diltiazem Hydrochloride: A Comparative Review,” Future Journal of Pharmaceutical Sciences 11 (2025): 95.

[45] B. R. Patil , O. G. Bhusnure , B. N. Paul , A. Y. Ghodke , and S. S. Mulaje , “Analytical Method Development and Validation for the Estimation of Diltiazem Hydrochloride in Bulk and Pharmaceutical Dosage Form by RP‐HPLC,” Journal of Drug Delivery and Therapeutics 14 (2024): 78.

[46] B. M. S. Kumar , B. Rajkamal , and B. Chandramowli , “A Validated RP‐HPLC Method for the Determination of Diltiazem in Raw Material and Pharmaceutical Dosage Form,” International Journal of Pharmacy and Biological Sciences 4 (2013): 580–588.

[47] H. Dash and S. K. Sahoo , “Method Development, Validation and Stability Study of Diltiazem by RP‐HPLC Method,” Journal of Pharmaceutical Research 8 (2014): 1528–1533.

[48] I. M. Samara , M. Ntorkou , C. I. Gioumouxouzis , C. Karavasili , P. D. Tzanavaras , and C. K. Zacharis , “Analytical QbD for the Optimization of a Multimode HPLC Method for the Investigation of Hydrochlorothiazide, Diltiazem and Propranolol Release from 3D Printed Formulation,” Journal of Pharmaceutical and Biomedical Analysis 248 (2024): 116324. 38924878 10.1016/j.jpba.2024.116324

[49] M. Shah , H. Kachhiya , J. Tandel , et al., “A Validated Liquid Chromatographic Method for Simultaneous Quantification of Lidocaine Hydrochloride and Diltiazem Hydrochloride,” Essential Chem 1 (2024): 1–7.

[50] S. Kumar , N. Singh , S. Satapathy , K. Barman , B. D. Kurmi , and P. Patel , “Green Chemistry‐Driven RP‐HPLC Method for Diltiazem and Lidocaine Hydrochloride in Bulk and Gel Formulations Using AQbD,” Accreditation and Quality Assurance 30 (2025): 605–623.

[51] F. Sadeghi , L. Navidpour , S. Bayat , and M. Afshar , “Validation and Uncertainty Estimation of an Ecofriendly and Stability‐Indicating HPLC Method for Determination of Diltiazem in Pharmaceutical Preparations,” Journal of Analytical Methods in Chemistry 2013 (2013): 353814. 24163778 10.1155/2013/353814 PMC3791576

[52] P. Usgaonkar , A. Adhyapak , and R. Koli , “Analytical Quality by Design (AQbD) Principles in Development and Validation of Stability‐Indicating RP‐HPLC Method for Simultaneous Estimation of Diltiazem HCl and Eugenol in Nanoethosomes,” Journal of Pharmaceutical Innovation 7, no. 11 (2024), 10.1002/sscp.202400155.

[53] F. G. K. Baucke , R. Neumann , and C. Alexander‐Weber , “Multiple‐Point Calibration with Linear Regression as a Proposed Standardization Procedure for High‐Precision pH Measurements,” Analytical Chemistry 65 (1993): 3244.

[54] S. Rondinini , P. R. Mussini , T. Mussini , and A. Vertova , “Definition of pH Scales, Standard Reference Values, Measurement of pH and Related Terminology,” Pure and Applied Chemistry 70 (1998): 1419.

[55] S. Rondinini , P. R. Mussini , and T. Mussini , “Standard pH Scale Definition,” Pure and Applied Chemistry 59 (1987): 1549–1560.

[56] P. H. Sherrod , NLREG Version 4.0 [software] (Sandia Software, 1991).

[57] E. G. Karageorgou , N. P. Kalogiouri , and V. F. Samanidou , “Green Approaches in High‐Performance Liquid Chromatography for Sustainable Food Analysis: Advances, Challenges, and Regulatory Perspectives,” Molecules 30 (2025): 3573. 40942098 10.3390/molecules30173573 PMC12430196

[58] P. Stepnowski , K. H. Blotevogel , P. Ganczarek , U. Fischer , and B. Jastorff , “Total Recycling of Chromatographic Solvents‐Applied Management of Methanol and Acetonitrile Waste,” Resources, Conservation, and Recycling 35 (2002): 163–175.

