# Supplementary material for: Sustainable Ethanol‐Based Reversed‐Phase Liquid Chromatography for Determination of Diltiazem pKa and Quantification with Integrated Green and White Analytical Metrics
Source: ChemistryOpen. 2026 Apr 20;15(5):e70215. doi: 10.1002/open.70215 (PMC13096578; doi:10.1002/open.70215)
Supplement: Supplementary file 1 — Supplementary Material [file OPEN-15-e70215-s001.pdf]

Supplementary material

Sustainable Ethanol-Based RPLC for Determination of Diltiazem pKa and Quantification with Integrated Green and White Analytical Metrics

Zehra ÜSTÜN[1*], İlkay KONÇE[2], Ebru ÇUBUK DEMİRALAY[3*]

[1]Suleyman Demirel University, Atayalvaç Vocational School of Health Services, Medical Services and Technical Department, 32400, Isparta, Turkey, 0000-0002-7061-6279
[2]Suleyman Demirel University, Faculty of Pharmacy, Department of Analytical Chemistry, 32260, Isparta, Turkey, 0000-0003-3542-7090
[3]Suleyman Demirel University, Faculty of Pharmacy, Department of Analytical Chemistry, 32260, Isparta, Turkey, 0000-0002-6270-7509

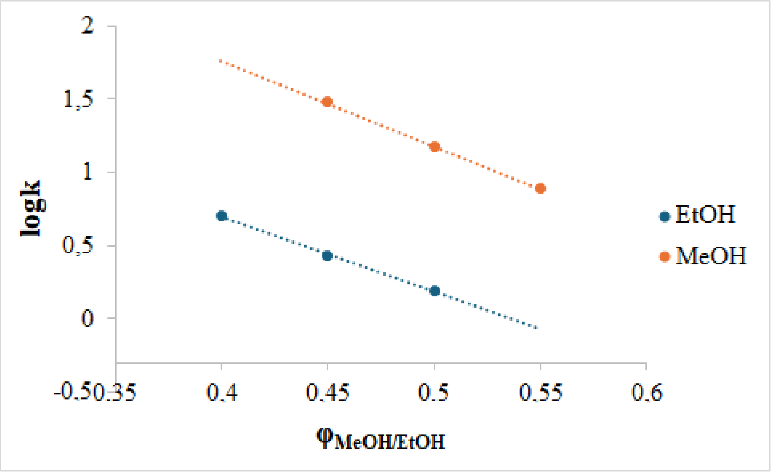

Fig. S1. logk-φ value relationship

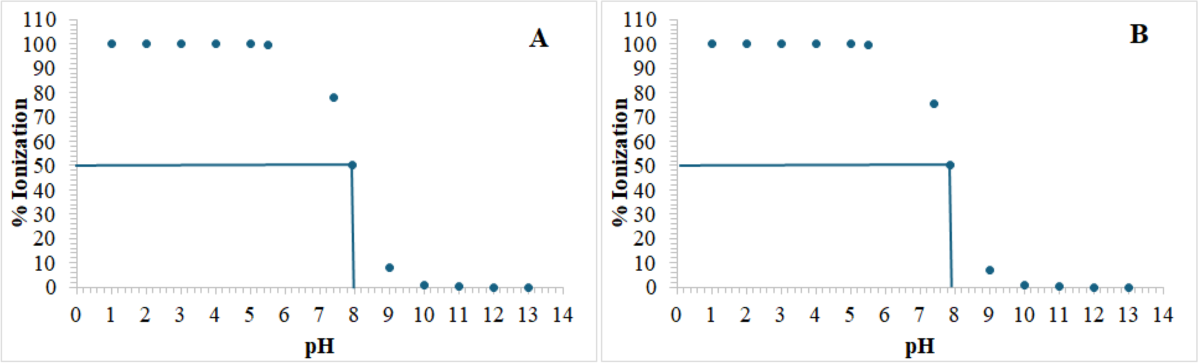

Fig. S2. Percentage ionization of studied compound as a function of pH A) EtOH-water B) MeOH-water binary mixtures

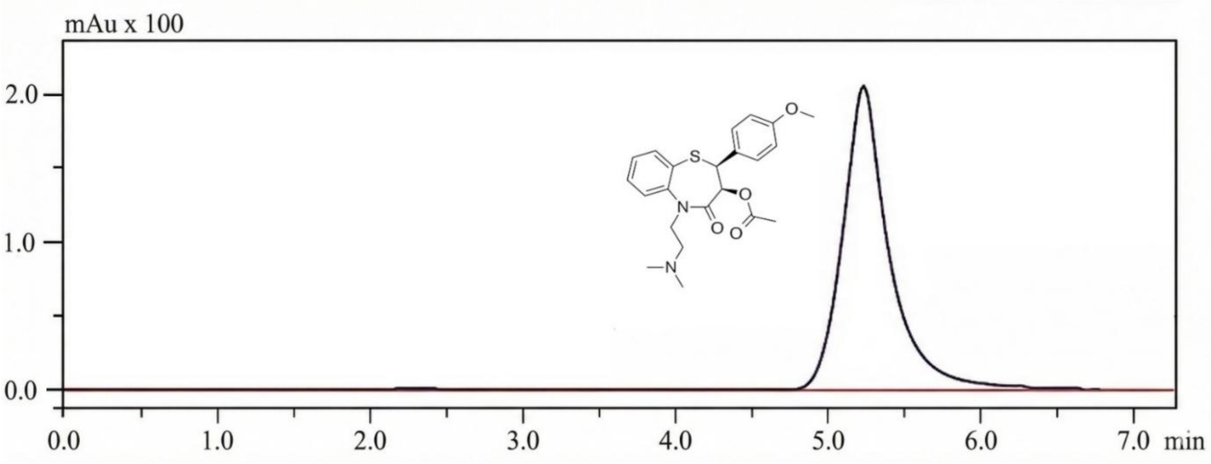

Fig. S3. Chromatogram of the diltiazem standard mixture in optimum chromatographic conditions.

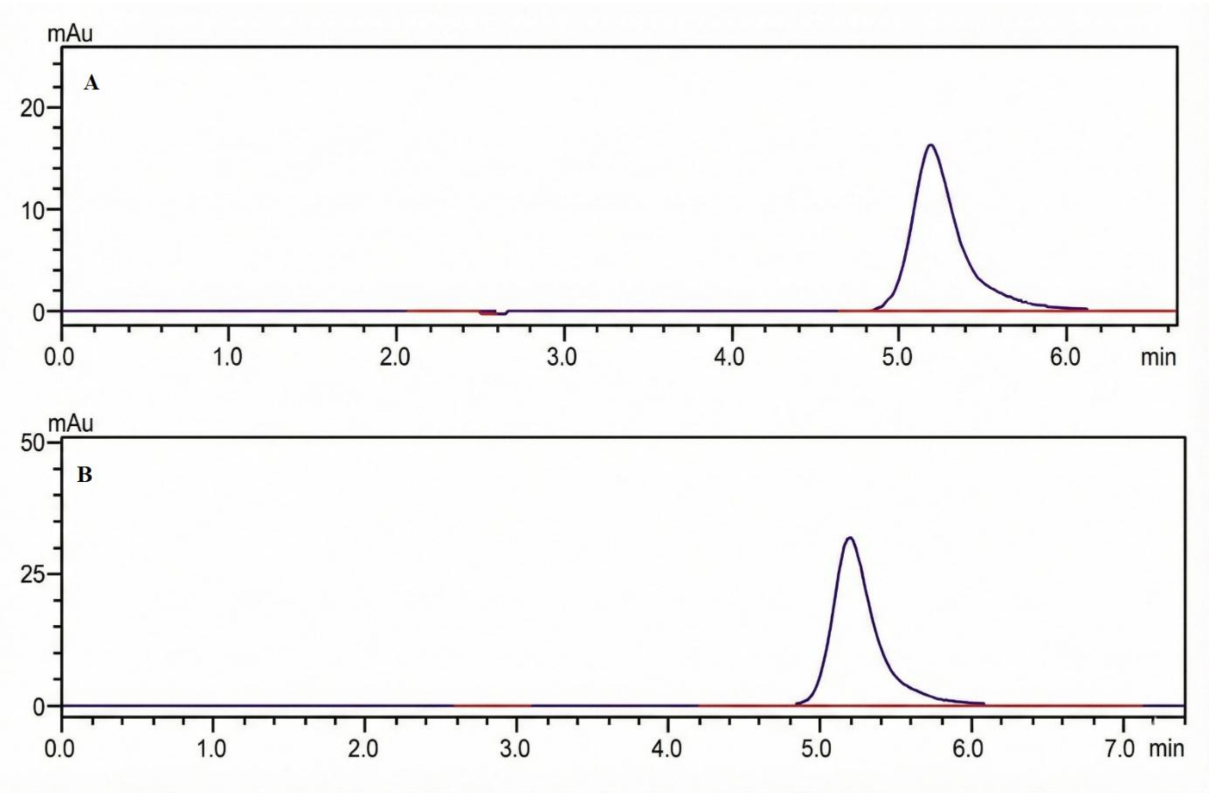

Fig. S4. A) Tablet sample analysis (diltiazem 5.0 µg/mL) B) Spiked sample analysis (diltiazem 5.0 µg/mL)

Table S1 Data obtained from the $\varphi_{solvent}$-logk relationship

$\varphi_{solvent}$	**Linear function**	**Correlation coefficient (r)**	**S**	k_w	φ_0
MeOH-water	logk = -5.830φ +4.097	0.999	5.830	12502.59	0.703
EtOH-water	logk = -5.130φ +2.749	0.9999	5.130	561.05	0.536

Table S2 Ionization percentage of the diltiazem at different pH values

**Medium**	**% Ionization value**				
	pH 2.0	pH 5.0	pH 7.4	pH 9.0	pH 12.0
**MeOH-water**	99.999	99.871	75.594	7.219	0.008
**EtOH-water**	99.999	99.887	77.894	8.131	0.009

Table S3 Intra and inter-day precision results for diltiazem

Concentration (µg/mL)	Intra-day mean (µg/mL)	%RSD (Intra-day)	Inter-day mean (µg/mL)	%RSD (Inter-day)
2	1.920	1.219	1.931	1.688
13	12.867	0.203	11.545	1.831

Table S4 The ruggedness results (analyst to analyst) for diltiazem

Replicate	Analyst 1	Analyst 2	Acceptance criteria
1	532352	537668	
2	532789	538411	
3	536487	539728	
4	532141	541233	
5	526952	534255	
**Pooled Mean**	535201.60		
**Pooled SD**	4088.54		
**Pooled RSD**	0.764		**≤ 3%**

Table S5 Robustness test results with different conditions

Parameter	Optimized condition	Used condition	Retention time (min)	Peak area (mAu)
**Mobile phase**	**40:60 (v/v%) EtOH-water binary mixture**	35:65(v/v%)	6.695	965139
		45:55 (v/v%)	3.822	974968
**pH**	**5.0**	4.0	4.587	976315
		6.0	7.088	971958
**Column temperature**	**37 ^{o}C**	25	5.701	987695
		45	4.876	989645
**Akış hızı**	**1 mL/min**	0.8	6.431	973707
		1.2	4.355	975655

Table S6 Applied stress conditions for forced degradation studies

Stress condition	Environment	Temperature	Exposure time (h)
**Acidic**	0.1 M HCl	25 °C	2, 6, 12, 24, 48
**Basic**	0.1 M NaOH	25 °C	2, 6, 12, 24, 48
**Oxidative**	3% H_2O_2	25 °C	2, 6, 12, 24, 48
**Photolytic**	UV light (365 nm)	Ambient	3, 6
**Thermal**	Dry heat	70 °C	3
